# Magnesium: Exploring Gender Differences in Its Health Impact and Dietary Intake

**DOI:** 10.3390/nu17132226

**Published:** 2025-07-04

**Authors:** Elisa Mazza, Samantha Maurotti, Yvelise Ferro, Alberto Castagna, Carmelo Pujia, Angela Sciacqua, Arturo Pujia, Tiziana Montalcini

**Affiliations:** 1 Department of Clinical and Experimental Medicine, University “Magna Græcia” of Catanzaro, 88100 Catanzaro, Italy; elisamazza@unicz.it (E.M.); tmontalcini@unicz.it (T.M.); 2 Department of Medical and Surgical Sciences, University “Magna Græcia” of Catanzaro, 88100 Catanzaro, Italy; yferro@unicz.it (Y.F.); a.castagna@unicz.it (A.C.); sciacqua@unicz.it (A.S.); pujia@unicz.it (A.P.); 3 O.U. Clinical Nutrition, Renato Dulbecco Hospital, 88100 Catanzaro, Italy; carmelopujia97@gmail.com; 4 Research Center for the Prevention and Treatment of Metabolic Diseases, University “Magna Græcia” of Catanzaro, 88100 Catanzaro, Italy

**Keywords:** magnesium, gender differences, sex-specific metabolism, magnesium bioavailability, hormonal regulation

## Abstract

**Background**: Magnesium (Mg^2+^) plays a fundamental role in various physiological processes, including neuromuscular function, glucose metabolism, cardiovascular regulation, and bone health. Despite its significance, the influence of sex on magnesium metabolism, requirements, and health outcomes remains unexplored. The aim of this review is to analyze sex-based differences in magnesium homeostasis, with a particular focus on hormonal regulation, body composition, and disease susceptibility. **Methods**: This narrative review, based on a non-systematic MEDLINE search conducted in January 2025, prioritized clinical trials from the past 15 years on human subjects and explored gender-specific aspects of magnesium intake, status, metabolism, and supplementation. **Results**: Hormonal fluctuations, particularly variations in estrogen levels, affect magnesium absorption, distribution, and retention, thereby influencing magnesium balance across different life stages such as puberty, pregnancy, and menopause. Additionally, dietary intake and lifestyle factors often differ between men and women, further impacting magnesium status. Emerging evidence suggests that suboptimal magnesium levels may differentially contribute to conditions such as osteoporosis, cardiovascular disease, and metabolic disorders in each sex. **Conclusions**: In conclusion, acknowledging sex-specific differences in magnesium metabolism is essential for developing personalized dietary guidelines and therapeutic strategies. Tailored nutritional approaches could significantly improve magnesium status, enhance overall health, and reduce the burden of chronic diseases linked to magnesium imbalance.

## 1. Introduction

In recent years, there has been a growing surge in interest in the influence of micronutrients on health, particularly their pivotal role in modulating key physiological and metabolic processes that exhibit pronounced gender-specific differences. Among these, magnesium has attracted considerable attention. Magnesium (Mg^2+^), a vital mineral, is a cofactor in over 600 enzymatic processes essential for maintaining physiological homeostasis [1]. These include muscle contraction, neuromuscular signaling, glucose metabolism, cardiac function, and blood pressure regulation [1,2,3]. Additionally, magnesium contributes significantly to cellular energy generation, ion transport across membranes, synthesis of genetic materials, and skeletal health [2,3]. Approximately 99% of the body’s total magnesium resides within cells, with 85% stored in bones, and only 1% in extracellular space. In the serum, around 70% of magnesium exists in its ionized (free) form, which plays a critical role in a wide range of physiological processes [2,4]. Extracellular magnesium is tightly regulated, with plasma levels consistently maintained between 1.6 and 2.3 mg/dL under normal physiological conditions in humans. Magnesium homeostasis relies on the interplay of three key organs: the intestine, responsible for absorbing dietary magnesium; the bones, which serve as the primary storage reservoir; and the kidneys, which control magnesium excretion [4]. The United States Food and Nutrition Board recommends a daily magnesium intake of 420 mg for adult males and 320 mg for adult females [1]. According to the European Food Safety Authority (EFSA), an Adequate Intake (AI) for magnesium is set at 350 mg/day for men and 300 mg/day for women [5]. Approximately 10% of this requirement is met through water consumption. Magnesium is abundant in green leafy vegetables, nuts, seeds, and unprocessed grains, with smaller amounts also found in fruits, fish, meat, and dairy products [3]. However, a significant portion of the population in Western countries fail to meet these recommended magnesium levels, primarily due to the widespread consumption of processed foods, demineralized water, and agricultural practices that rely on magnesium-deficient soils [1,6]. Magnesium has garnered increasing attention among researchers in recent years due to its role in numerous physiological processes and its potential therapeutic implications in addressing a wide range of chronic diseases [3,7,8]. Despite its importance, magnesium deficiency is prevalent worldwide and has been linked to a range of chronic diseases, such as hypertension [9], diabetes [10], coronary heart disease [11,12,13], and osteoporosis [13,14].

Recent research underscores the gender-specific roles of nutrients, highlighting the need to consider sex-related physiological, hormonal, and lifestyle differences when formulating dietary recommendations and supplementation strategies to optimize health outcomes [15,16]. Despite magnesium’s well-established role in numerous physiological processes, its metabolism, requirements, and health effects have traditionally been studied without accounting for potential sex differences.

While the physiological significance of magnesium is well established, gender-specific differences in its metabolism remain largely unexplored. This review aims to elucidate the metabolic effects of magnesium from a gender-specific perspective, with a focus on the underlying mechanisms and their implications for health. Hormonal fluctuations, body composition, and disease susceptibility, which vary significantly between sexes, substantially influence the absorption, distribution, and balance of magnesium. Understanding how these factors interact is crucial for developing more targeted nutritional and therapeutic strategies to optimize clinical outcomes. This study seeks to address these gaps by analyzing how sex-based variations affect magnesium metabolism, providing new insights to refine dietary recommendations and clinical management strategies. Exploring these aspects is critical to ensuring that nutritional guidelines and therapeutic interventions are truly effective and personalized, reflecting the specific needs of both men and women.

## 2. Search Methods

This narrative review did not involve a systematic literature search. However, a query was conducted on MEDLINE via PubMed in January 2025, using a combination of the following keywords: “Magnesium”, “Magnesium intake”, “Magnesium deficiency”, “Magnesium metabolism”, “Magnesium status”, “Magnesium absorption”, “Magnesium supplementation”, “Health”, “Dietary patterns”, “Gender”, “Gender differences”, “Gender-specific nutritional needs”, “female”, “male”, “micronutrients”, “women”, and “men.” The search strategy included Boolean operators such as AND to combine terms. Examples of the search combinations used are as follows: “Magnesium intake” AND “Gender” AND “Micronutrients”, “Magnesium intake” AND “Women” AND “Men” AND “Micronutrients”, “Magnesium status” AND “Gender differences”, “Magnesium supplementation” AND “Health” AND “Women”.

No date restrictions were applied. Priority was given to clinical trials published in the past 15 years, written in English, and conducted on human subjects. The retrieved studies were carefully screened for relevance to the focus of the review.

## 3. General Effects of Magnesium on Hormonal Balance and Metabolism

Magnesium is essential for a wide range of physiological functions, including energy metabolism, neuromuscular coordination, and cardiovascular regulation [17]. It plays a crucial role in enzymatic reactions, supports DNA and protein synthesis, and helps control oxidative stress and inflammation [8,17]. In bone physiology, magnesium contributes to skeletal development by modulating osteoblast and osteoclast activity, regulating calcium and phosphate homeostasis, and influencing the structural integrity of the bone matrix [14]. It acts as a cofactor for enzymes involved in vitamin D activation and parathyroid hormone (PTH) signaling, both essential for mineral balance [18]. Magnesium also regulates the expression of key osteogenic markers such as *Runx2*, *Osterix* (*SP7*), and *osteocalcin* (*BGLAP*) in osteoblasts [19], while influencing the RANK/RANKL/OPG system to control osteoclastogenesis [20]. In addition to these well-recognized functions, magnesium is increasingly studied for its involvement in hormonal regulation and metabolic processes [17], particularly its effects on thyroid activity, stress response, and insulin sensitivity. Although sex-related hormonal differences can modulate magnesium metabolism, the underlying mechanisms rely on shared physiological pathways.

### 3.1. Magnesium and Thyroid Hormone Regulation

Thyroid hormones play a fundamental role in regulating basal metabolic rate (BMR) and thermogenesis by enhancing mitochondrial metabolism, which stimulates mitochondrial respiration and oxidative phosphorylation [21]. Magnesium is essential for these processes, as it contributes to the regulation of thyroid hormone functions. It plays a crucial role in energy-dependent reactions and ATP generation, directly or indirectly supporting multiple enzymatic processes [22].

Magnesium is involved in thyroid hormone synthesis by facilitating iodide absorption and the deiodination process necessary for hormone activation. Specifically, it supports the function of enzymes such as iodothyronine deiodinase, which require magnesium as a cofactor to drive reduction processes in the electron transport chain [21,22]. Studies indicate that magnesium availability influences thyroid hormone activity, contributing to the fine-tuning of metabolic regulation.

Throughout different life stages, thyroid hormone levels fluctuate, with variations observed during perimenopause and postmenopause in women [21]. Animal studies have shown that magnesium supplementation significantly increases the uptake of radioactive iodine by thyroid cells, especially under certain pathological conditions. These hormonal changes may, in turn, affect magnesium homeostasis, highlighting possible sex-specific differences in thyroid function. Recent evidence suggests that magnesium levels tend to decrease during perimenopause and postmenopause, while thyroid-stimulating hormone (TSH) levels show concurrent adjustments [23]. Similarly, thyroid function and magnesium metabolism are interrelated in men, with variations observed across different physiological conditions.

Ensuring adequate magnesium intake is important for supporting thyroid function and maintaining metabolic balance. Future research should continue exploring the interplay between magnesium and thyroid hormones to further understand its role in energy metabolism and physiological adaptations across life stages.

### 3.2. Magnesium on Cortisol and Stress Regulation

Over the years, accumulating evidence has highlighted magnesium’s essential role in regulating several key physiological pathways involved in the body’s response to stress [24]. Magnesium directly influences cortisol regulation by modulating the hypothalamic–pituitary–adrenal (HPA) axis, a critical system responsible for the release of cortisol in response to stress [25]. At the molecular level, magnesium modulates N-methyl-D-aspartate (NMDA) receptors, thereby reducing excitatory glutamatergic neurotransmission in the hypothalamus, which helps attenuate the activation of corticotropin-releasing hormone (CRH) and adrenocorticotropic hormone (ACTH) pathways [26].

By maintaining adequate magnesium levels, cortisol homeostasis occurs, preventing excessive release that can lead to mental and physical health issues such as anxiety, sleep disturbances, and metabolic dysfunction [24,27].

Moreover, chronic cortisol elevation contributes to inflammation by increasing pro-inflammatory cytokines such as *TNF*-α, *IL-1β*, and *IL-6* and increases reactive oxygen species (ROS) production, which can amplify the harmful effects of stress [24]. A study investigating magnesium supplementation in individuals experiencing chronic emotional stress found that most participants exhibited symptoms such as irritability, fatigue, and sleep disturbances, with a significant proportion showing magnesium deficiency at baseline [28]. Therefore, maintaining adequate magnesium levels is especially important, as it helps mitigate the negative effects of stress on overall health, particularly in relation to hormonal fluctuations and emotional well-being.

### 3.3. Magnesium’s Role in Insulin Sensitivity, Glucose Homeostasis and Vascular Health

Magnesium is a key modulator of insulin signaling, influencing both its secretion and action, which are fundamental in the regulation of glucose homeostasis [29]. This mineral supports the activity of the insulin receptor’s tyrosine kinase and its autophosphorylation, crucial mechanisms for maintaining insulin responsiveness. Specifically, magnesium promotes the phosphorylation of the insulin receptor β-subunit and facilitates the recruitment of insulin receptor substrate-1 (IRS-1), activating the PI3K/Akt pathway that drives GLUT4 translocation for glucose uptake [30].

Physiological differences in body composition and hormonal profiles between genders can affect magnesium status and glucose metabolism. For instance, in women, hormonal fluctuations and differences in adipose tissue distribution may impact magnesium levels, modulating insulin receptor activity and glucose uptake [31].

Magnesium contributes to glucose homeostasis by fine-tuning insulin receptor activity and glucose uptake mechanisms [32]. Maintaining adequate magnesium intake is particularly important in both men and women, as it helps support insulin efficiency, sustain glucose regulation, and promote energy metabolism.

A systematic review and meta-analysis of double-blind randomized trials found that magnesium intake improved fasting glucose and 2 h glucose tolerance in healthy individuals and those with metabolic alterations. Additionally, magnesium status was associated with enhanced markers of insulin sensitivity [33]. Magnesium facilitates insulin sensitivity by modulating the tyrosine kinase activity of the insulin receptor and promoting autophosphorylation of its β-subunit, leading to efficient downstream signaling [34].

Furthermore, magnesium regulates calcium influx by blocking L-type calcium channels in adipocytes. This action helps maintain intracellular calcium at physiologically appropriate levels, preserving insulin receptor conformation and preventing calcium-mediated inhibition of insulin signaling cascades, including IRS-1 and Akt [27].

Under conditions of magnesium deficiency, altered calcium flux may impair insulin receptor function, contributing to suboptimal glucose uptake [35]. Similarly, magnesium inhibits the NMDA receptor in neurons, and adequate magnesium availability helps prevent excessive calcium accumulation and unnecessary activation of inflammatory pathways [34].

Given its essential role in insulin regulation, maintaining adequate magnesium levels supports metabolic homeostasis and optimizes glucose utilization. Further research is needed to delineate the mechanistic interactions between magnesium status and insulin dynamics, particularly in response to age-related metabolic adaptations.

Magnesium plays a crucial role in supporting vascular health by enhancing endothelium-dependent vasodilation, improving lipid profiles, and exerting antihypertensive and anti-inflammatory effects through its function as a natural calcium channel blocker [36,37].

At the cellular level, magnesium enhances Nitric Oxide (NO) production via endothelial nitric oxide synthase (eNOS) activation and reduces vascular tone by inhibiting calcium influx in smooth muscle cells [38].

A meta-analysis has indicated that higher dietary magnesium intake is associated with better blood pressure regulation in men [39] while a cross-sectional study demonstrated that lower magnesium levels are linked to unfavorable variations in lipid metabolism and blood pressure regulation (OR: 2.8 for dyslipidemia and OR: 1.9 for hypertension) [40]. Moreover, several meta-analyses have consistently reported a dose-dependent protective effect of magnesium on stroke risk, with those consuming the highest amounts exhibiting a 22% lower risk of stroke compared to those with the lowest intakes [41,42]. These findings highlight the importance of optimizing magnesium intake in men, not only for metabolic and musculoskeletal health but also to support cardiovascular function.

## 4. Magnesium Deficiency: Epidemiological Data and Health Implications

In modern societies, inadequate magnesium intake is common, with average consumption often falling below the recommended level for adolescents and adults [43]. Magnesium deficiency is observed more frequently in women than in men [44,45]. This discrepancy may be partly attributable to estrogen’s role in enhancing tissue magnesium utilization, suggesting that hormonal fluctuations in women may influence and regulate magnesium homeostasis [3]. Nevertheless, suboptimal magnesium status is also a concern among men. Although epidemiological data indicate that the prevalence of subclinical magnesium deficiency is generally lower in men, a significant proportion, particularly among older individuals and those with high physical activity levels, exhibit inadequate magnesium intake [46]. In these groups, factors such as decreased intestinal absorption, increased urinary excretion, and dietary imbalances contribute to a state of subclinical magnesium deficiency, potentially affecting muscle function, cardiovascular regulation, and metabolic health [1,6,47]. In addition to the prevalence data, evidence suggests that suboptimal magnesium status in men can adversely affect cardiovascular function.

This insufficiency is largely attributed to magnesium loss during food refining processes [48], resulting in subclinical magnesium deficiency. The prevalence of subclinical magnesium deficiency ranges from 2.5% to 15% among generally healthy women and reaches approximately 20% in young women aged 18 to 22 years [49]. An increasing body of evidence suggests that magnesium deficiency significantly contributes to various physiological conditions across a woman’s lifespan, posing substantial risks to health and overall quality of life [50]. The optimal dietary calcium-to-magnesium ratio is approximately 2:1 and exceeding this ratio has been associated with a higher risk of metabolic, inflammatory, and cardiovascular disorders [6]. The balance between calcium and magnesium intake is essential for maintaining bone health and metabolic functions. An imbalance, particularly an excessive calcium-to-magnesium ratio, can not only impair magnesium absorption but also exacerbate inflammation, which is a risk factor for several chronic conditions [51]. Notably, excessive calcium intake can hinder magnesium absorption, thus increasing magnesium requirements and potentially resulting in subclinical magnesium deficiency [1,6].

## 5. Magnesium and Gender Differences: Physiological and Metabolic Implications

While magnesium’s physiological roles are well recognized, emerging evidence suggests that its metabolism, requirements, and health impacts may differ significantly between men and women. Gender-specific differences in the metabolic effects of magnesium are shaped by hormonal variations, life stage transitions, and lifestyle factors. These differences are particularly evident under conditions like reproductive health in women and testosterone regulation in men, which can have profound effects on health outcomes. This section explores the gender-specific roles of magnesium, highlighting its unique contributions to health and disease in men and women.

### 5.1. Magnesium and Women’s Physiology: A Key Nutrient in Health

**Figure 1:**
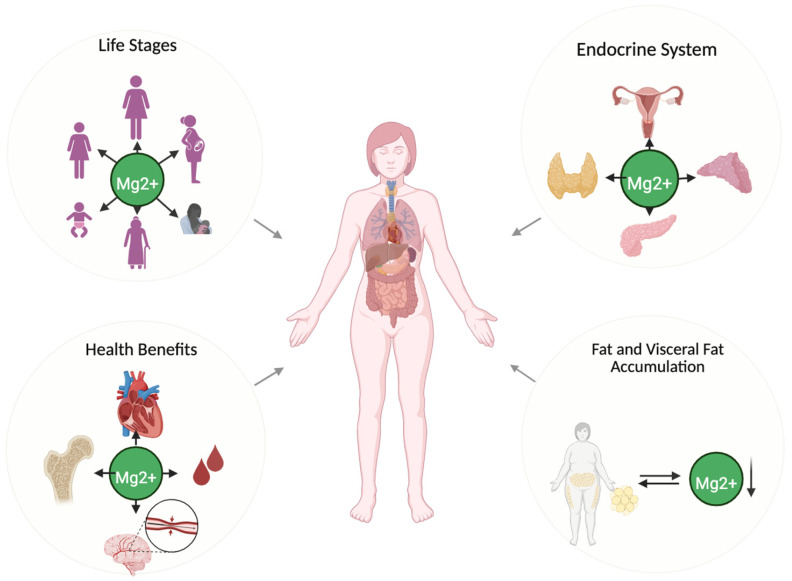
The essential role of magnesium throughout a woman’s life. Role of magnesium (Mg^2+^) in female health across different life stages. Magnesium plays a crucial role in endocrine system regulation, visceral fat accumulation, and various health benefits, including cardiovascular, bone, and neurological functions. The downward arrow indicates low magnesium levels. Created in BioRender. https://BioRender.com/.

Recent research has expanded our understanding of magnesium’s role in women’s health, highlighting its interactions with key physiological processes. This section focuses on how magnesium influences hormonal balance, metabolic regulation, and body composition, with implications for overall well-being (Figure 1).

Women’s energy and nutritional requirements undergo significant changes throughout different life stages, driven by substantial hormonal fluctuations from puberty to reproductive age, through the climacteric period, and into postmenopause [43,50]. During pregnancy and lactation, this mineral plays a fundamental role in cellular function, supporting maternal and fetal health by promoting cell division, protein synthesis, and metabolic homeostasis. Ensuring adequate intake during these stages optimizes physiological adaptation and supports maternal well-being [52].

Throughout a woman’s life, magnesium plays a role in multiple physiological processes. From reproductive age onward, hormonal regulation is influenced by factors such as oral contraceptive use, which affects serum magnesium levels in millions of European women (≈22 million) [50]. As women approach perimenopause, maintaining optimal magnesium levels supports metabolic stability and inflammatory modulation [12].

Magnesium contributes to cellular redox balance and regulates inflammatory responses, as it is involved in pathways controlling cytokine activity and C-reactive protein levels [53]. The calcium-to-magnesium ratio is also relevant for modulating homeostatic processes linked to reproductive health [54,55]. Magnesium plays a role in neurotransmitter regulation, supporting mood stability and neuromodulation [56]. Additionally, its involvement in muscle relaxation contributes to uterine contractility regulation, potentially influencing menstrual comfort [54,57]. Through its impact on hormonal and neuromuscular regulation, magnesium supports physiological adaptations across different phases of the menstrual cycle [58,59].

These combined effects highlight the importance of magnesium in supporting women’s well-being and quality of life throughout hormonal fluctuations and the menstrual cycle [60].

#### 5.1.1. Menstrual Cycle and Premenstrual Syndrome (PMS): Magnesium’s Role in Symptom Modulation

Magnesium plays a vital role in maintaining hormonal equilibrium and modulating inflammatory responses throughout the menstrual cycle [3]. The menstrual cycle represents a significant aspect of female physiology, characterized by cyclical hormonal changes that influence metabolic and neuromuscular function [60]. In the follicular phase, it may help regulate estrogen-driven inflammatory pathways, while in the luteal phase, it contributes to muscle relaxation and electrolyte balance, potentially alleviating premenstrual symptoms such as cramps and bloating [61]. This mineral also supports glucose homeostasis and neurotransmitter function, playing a role in mood stability [62]. Given its impact on hormone regulation, magnesium is involved in cyclical metabolic adjustments.

PMS is a physiological response to cyclical hormonal variations, influencing mood, energy levels, and neuromuscular function [63]. It occurs during the luteal phase of the menstrual cycle and is characterized by the transient presence of physical and behavioral symptoms, which resolve within a few days following menstruation [64,65]. The physiological mechanisms underlying PMS are not yet fully understood, but proposed hypotheses involve hormonal fluctuations, micronutrient dynamics (such as magnesium and vitamin B6), and genetic predisposition [64,66,67]. Magnesium’s role in supporting PMS-related metabolic adaptations is linked to its ability to modulate progesterone interactions with the central nervous system, potentially contributing to mood stabilization and neuromuscular homeostasis [68]. While the exact pathways remain under investigation, magnesium appears to influence neurotransmitter signaling and neuroendocrine responses [69]. Magnesium, alone or in combination with vitamin B6, has been studied for its potential role in modulating menstrual cycle-related symptoms. This strategy is supported by evidence showing that women with PMS have lower magnesium levels compared to healthy women [70]. The exact mechanism by which magnesium alleviates PMS symptoms remains unclear, but several hypotheses have been suggested. Notably, the most significant improvements are typically observed in emotional and behavioral symptoms, such as depression, irritability, fatigue, and anxiety. It is proposed that magnesium may primarily exert its effects by normalizing the actions of various hormones, particularly progesterone, on the central nervous system [68]. Several studies have explored the potential benefits of magnesium supplementation in supporting menstrual function. Facchinetti et al. [67] conducted a clinical trial in which magnesium intake significantly improved PMS symptoms, as assessed by a standardized questionnaire, compared to placebo. Similar findings were reported by Walker et al. [69] and Quaranta et al. [68], suggesting that optimal magnesium levels may contribute to menstrual well-being over time. A more recent 2024 study by Yaralizadeh et al. [71] confirmed these findings, showing that magnesium supplementation helped maintain physiological balance during the menstrual cycle, with a dose-dependent effect on symptom reduction.

Dysmenorrhea is characterized by cyclic uterine contractility changes, influenced by hormonal and metabolic factors [71]. Prostaglandin-mediated mechanisms contribute to variations in smooth muscle tone, impacting menstrual physiology [71,72]. Non-pharmacological strategies, including magnesium intake, have been explored for their role in neuromuscular modulation [73,74].

Clinical studies suggest that magnesium may act as a calcium antagonist, contributing to the regulation of uterine contractility. Findings from controlled trials indicate that magnesium supplementation supports neuromuscular relaxation, with effects observed over several months of consistent intake [75,76,77]. These results highlight magnesium’s potential role in optimizing menstrual physiology.

Hormonal fluctuations across the menstrual cycle affect neurotransmitter dynamics, influencing neuronal excitability and metabolic activity [78]. Menstrual migraines, often associated with estrogen fluctuations, involve complex neurovascular interactions. This mineral has been studied for its potential role in supporting neural homeostasis during the menstrual cycle [78,79]. It is linked to estrogen withdrawal, which influences neuronal activity and receptor density. Menstrual migraines, which may occur with or without aura, are typically more severe, recurrent, and less responsive to treatment than non-menstrual migraines. Acute treatments include NSAIDs, triptans, and ergot derivatives, sometimes combined with antiemetics. Preventive options, though less studied, may involve perimenstrual estrogen, short-term NSAIDs, or triptans [79]. A placebo-controlled trial by Peikert et al. [80] reported a 41.6% improvement in migraine frequency with magnesium supplementation, compared to 15.8% with placebo. While findings are not universally consistent, magnesium’s role in neurotransmitter modulation is well-documented [80,81]. Stress-related mechanisms may further influence migraine susceptibility, particularly during the perimenstrual period, when endocrine and neurochemical thresholds are dynamic [82,83].

#### 5.1.2. Magnesium, Body Composition, and Visceral Fat Regulation in Women

Hormonal profiles influence metabolic regulation and body composition, with gender-specific patterns in adipose tissue distribution [15,16]. Women generally accumulate higher subcutaneous fat, while hormonal fluctuations, especially during menopause, are associated with shifts in visceral adiposity [84]. These metabolic adjustments modulate nutrient utilization and inflammatory responses, influencing long-term physiological adaptations [15].

Magnesium plays a key role in metabolic homeostasis, and evidence suggests that body composition may correlate with magnesium levels [85]. Studies indicate that women with increased adiposity may exhibit lower magnesium status, possibly reflecting metabolic demands [46]. A meta-analysis of 32 randomized controlled trials found that magnesium supplementation supported BMI regulation, particularly in populations with altered metabolic efficiency. The most pronounced effects were observed in women, highlighting a potential link between magnesium intake and metabolic resilience [86]. Additionally, chronic low-grade inflammation associated with adiposity may be influenced by magnesium homeostasis. Magnesium is involved in the regulation of inflammatory pathways and oxidative stress, which are relevant to overall metabolic function [27]. This interplay suggests that magnesium status may contribute to individual variations in fat metabolism, emphasizing the importance of adequate intake for maintaining metabolic equilibrium.

### 5.2. Magnesium and Male Physiology: Roles and Health Implications

**Figure 2:**
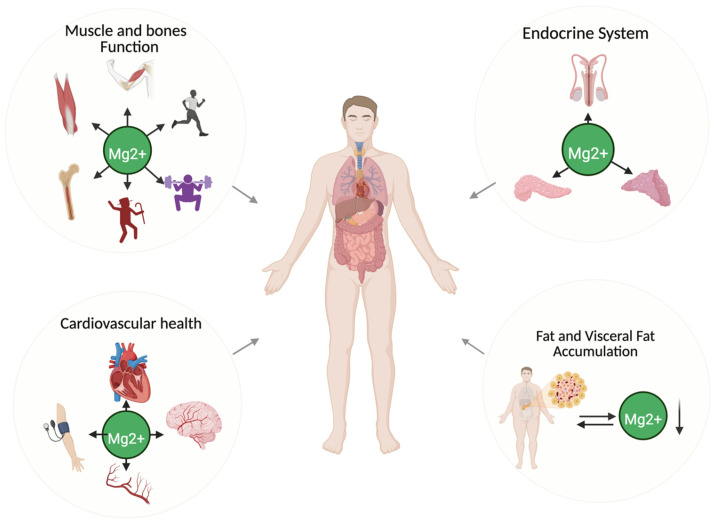
The essential role of magnesium throughout a male’s life. Role of magnesium (Mg^2+^) in male health. Magnesium contributes to endocrine system regulation, cardiovascular health, muscle and bone function, and the modulation of visceral fat accumulation. The downward arrow indicates low magnesium levels. Created in BioRender. https://BioRender.com/.

Emerging research continues to underscore the role of magnesium in men’s health, particularly in areas such as hormonal regulation, inflammatory modulation, and metabolic efficiency. However, studies focusing specifically on men remain comparatively limited. This section explores magnesium’s physiological relevance in male health, with an emphasis on hormonal balance, muscle function, and body composition (Figure 2).

Magnesium is involved in numerous biological processes, including bone health, muscle function, cardiovascular regulation, and metabolism, all of which are integral to male physiology [8]. Evidence suggests that men typically have higher serum magnesium concentrations compared to women [87].

Magnesium plays a key role in muscle function, supporting both contraction and relaxation, which is essential for physical performance, particularly in active men and athletes [88]. Additionally, this mineral is fundamental for maintaining bone integrity in aging men, aiding bone mineralization and supporting skeletal structure, which contributes to overall mobility and strength [89].

Beyond musculoskeletal health, magnesium contributes to cardiovascular function by regulating blood pressure and vascular tone. Evidence suggests that adequate magnesium intake is particularly beneficial for maintaining vascular health and promoting optimal endothelial function [35,90,91]. Furthermore, magnesium supports metabolic balance by facilitating insulin sensitivity and glucose regulation, especially in men with higher body weight or metabolic demands [92].

Another significant aspect of magnesium’s role is its interaction with anabolic hormones, particularly testosterone, which is essential for muscle maintenance, metabolic efficiency, and overall vitality. Research indicates that sufficient magnesium intake supports healthy testosterone levels, particularly in physically active men or those experiencing age-related hormonal changes [93].

#### 5.2.1. Magnesium on Sex Hormones and Testosterone Levels

Magnesium plays a significant role in modulating anabolic hormones in men, particularly testosterone. As the primary male sex steroid, testosterone is predominantly synthesized (approximately 95%) by Leydig cells in the testes, with the remaining 5% derived from the peripheral conversion of adrenal androgens [94]. Magnesium contributes to testosterone regulation [77], with research indicating a positive correlation between magnesium status and both free and total testosterone concentrations [93,95]. Testosterone is essential for maintaining lean body mass, muscle strength, and metabolic function. Studies have demonstrated that magnesium supplementation is associated with increased free and total testosterone levels, with more pronounced effects observed in individuals engaging in physical activity [95]. Furthermore, Maggio et al. [93] found that in aging men, magnesium levels were strongly and independently associated with total testosterone and insulin-like growth factor 1 (IGF-1), both of which are critical for preserving anabolic function. The mechanisms underlying this relationship may involve magnesium’s influence on the hypothalamic–pituitary–gonadal axis and its role in mitigating oxidative stress, which can impact hormone production and metabolic regulation [93,96]. Ensuring optimal magnesium intake, along with regular physical activity, may be crucial for maintaining hormonal balance in men.

#### 5.2.2. Muscle Mass in Men

Magnesium is one of the most extensively studied minerals in relation to muscle physiology. Its effects on skeletal muscle and physical performance stem from its fundamental role in energy metabolism, including phosphorylation processes, ATP-dependent reactions, and intracellular transport, all of which are essential for muscle contraction and relaxation [47,97]. This mineral supports oxygen utilization during exercise and endurance performance [98].

Research highlights its contribution to muscle health and function across different life stages [88]. Studies have shown that magnesium is involved in maintaining mitochondrial efficiency, reducing oxidative stress, and supporting neuromuscular function [95,99]. Evidence suggests that insufficient intracellular magnesium may compromise mitochondrial energy production, increase reactive oxygen species (ROS) generation, and lead to structural and functional disruptions in muscle cells [100,101]. Additionally, magnesium levels are influenced by systemic inflammation, a condition associated with increased ROS production and reduced expression of antioxidant enzymes, which can negatively impact muscle function [102]. Various factors affect magnesium levels and anabolic hormone regulation in adult and elderly men, with inflammation being a key modulator of both. Given that elevated inflammatory markers and declining anabolic factors are associated with muscle loss and reduced physical performance, strategies involving adequate magnesium intake may hold potential for optimizing muscle function [103].

#### 5.2.3. Fat Mass and Visceral Fat

Gender differences in body composition are well documented, with men typically accumulating a higher proportion of abdominal visceral fat compared to women. This distribution pattern influences metabolic regulation and cardiovascular risk [104,105]. Preclinical studies suggest a potential role for magnesium in modulating body composition, though human studies specifically examining magnesium intake and fat distribution remain limited [106]. However, observational data indicate an inverse association between dietary magnesium intake and waist circumference in men, suggesting a potential link between magnesium levels and abdominal fat regulation [107]. In clinical settings, studies have reported a negative correlation between magnesium intake and adiposity markers in male populations. Specifically, a 10 mg increase in magnesium intake per 1000 kcal/day was associated with a 0.72% reduction in BMI and a 0.49 cm decrease in waist circumference [87]. These findings suggest that adequate magnesium intake may play a role in metabolic efficiency and body composition maintenance.

Unlike women, who tend to store more subcutaneous fat, men exhibit a preferential accumulation of visceral adipose tissue, which is more metabolically active and sensitive to hormonal influences such as testosterone and cortisol. Given its role in metabolic regulation, magnesium may influence this distribution by modulating insulin sensitivity and lipid metabolism, as previously discussed. Additionally, men generally have a higher resting energy expenditure and fat oxidation rate than women, factors that could affect the physiological demand for magnesium in energy homeostasis [27,107]. Magnesium’s antioxidant properties, mediated through key enzymatic pathways, also play a role in fat metabolism and tissue distribution, particularly in abdominal fat accumulation [27,107]. Evidence suggests that magnesium status affects insulin sensitivity, inflammatory markers, and energy homeostasis—critical factors in visceral fat regulation [27,37,108]. These processes collectively influence metabolic health in men, reinforcing the importance of optimal magnesium intake in maintaining body composition and cardiovascular function.

Table 1 summarizes the key gender-specific differences in magnesium metabolism, physiological roles, and health outcomes discussed in this section.

## 6. Strategies for Enhancing Magnesium Status and Preventing Deficiency

### 6.1. Dietary Sources of Magnesium and Gender-Specific Needs

Magnesium is a vital nutrient that must be acquired through one’s diet to meet recommended intake levels and prevent deficiencies (Table 2). Achieving optimal magnesium levels requires not only identifying dietary sources but also understanding factors that influence its bioavailability, absorption, and excretion. These factors can vary significantly across genders due to hormonal differences, dietary habits, and physiological demands. Gaining deeper insights into its dietary sources, bioavailability, and the factors regulating its absorption and excretion, is particularly important when addressing gender-specific nutritional needs.

#### Magnesium-Rich Foods and Dietary Sources

Magnesium is naturally present in a wide range of plant- and animal-based foods, as well as in beverages. Excellent sources include green leafy vegetables (e.g., spinach), legumes, nuts, seeds, and whole grains, with fiber-rich foods generally offering higher magnesium content [113] (Table 3). Some fortified products, such as certain breakfast cereals, also contribute to dietary intake. However, food processing methods like grain refining, which remove the nutrient-dense germ and bran, can significantly deplete magnesium levels [113]. Water, whether tap, mineral, or bottled, can also provide magnesium, with concentrations varying widely from as low as 1 mg/L to over 120 mg/L, depending on the source and brand [115]. On average, the body absorbs 30% to 40% of the magnesium consumed through dietary intake [116]. However, dietary patterns and food choices often differ between men and women, influencing the overall magnesium status. Men generally consume more magnesium-rich foods due to larger portion sizes and higher caloric needs, while women often struggle to meet magnesium requirements, particularly during life stages like menopause, pregnancy, and lactation, when physiological demands are heightened [117,118].

### 6.2. Magnesium and Factors Influencing Bioavailability

Magnesium is widely present in foods, though its content is influenced by factors such as soil quality, irrigation water, fertilizers, preservation methods, and food processing techniques [17]. Acidic, light, and sandy soils are typically low in magnesium. Additionally, agricultural practices, such as high-concentration potassium and ammonium fertilizers, deplete magnesium levels in crops [17,120]. As previously mentioned, food processing methods like boiling vegetables and refining grains, which remove the germ and bran, lead to substantial reductions in magnesium content. The magnesium loss during food refinement is significant: 82% in white flour, 83% in polished rice, 97% in starch, and 99% in white sugar [17].

Beyond food sources, magnesium bioavailability is modulated by intrinsic physiological factors, including sex, age, and hormonal status. Estrogen positively influences magnesium absorption and retention, but levels decline after menopause, potentially increasing the risk of deficiency in women [109]. Men, on the other hand, generally exhibit stable magnesium absorption but may experience higher excretion rates due to lifestyle factors such as high alcohol consumption [121].

With aging, intestinal absorption of magnesium decreases while renal excretion increases [122]. Evidence indicates that magnesium deficiency is linked to cellular senescence and an accelerated aging phenotype [123]. Given magnesium’s essential role in muscle function, maintaining adequate intake may support mobility and musculoskeletal health in older adults [16,124]. These changes are particularly observed in postmenopausal women and may influence overall musculoskeletal health [16]. Lower magnesium intake and absorption have been observed in older adults, potentially influenced by hormonal changes, including reduced estrogen levels, which may affect magnesium excretion [125]. Lo Piano et al. [126] highlighted the risks and consequences associated with decreased magnesium intake and absorption in older adults. There is growing evidence supporting the beneficial role of magnesium in bone health in older adults. Erem et al. [110] reviewed studies suggesting that magnesium intake plays a role in bone mineralization and skeletal integrity in older adults. Magnesium contributes to bone metabolism, influencing calcium balance and skeletal maintenance. Furthermore, the prospective Kuopio Ischemic Heart Disease study in Japan found that low serum magnesium concentrations in men aged 42 to 61 were associated with an increased risk of bone fractures [127]. This further underscores the critical role of magnesium in bone health. As with the previously noted impact of magnesium intake on bone mineralization, growing evidence suggests an association between magnesium and the preservation and functionality of skeletal muscle. Welch et al. [111] analyzed data from 56,575 individuals (39–72 years) in the UK Biobank, finding positive associations between magnesium intake, grip strength (*p* trend < 0.001), and FFM% (*p* trend < 0.001). The link with grip strength was stronger in men ≥ 60 years, while the opposite was true for women. This is the largest study to date on magnesium intake and skeletal muscle functionality. Finally, increased magnesium intake has been associated with better functional mobility, particularly in relation to gait speed, in older adults living in the community [128]. This suggests that ensuring sufficient intake of this essential mineral may contribute to the prevention of unhealthy aging.

Emerging evidence also points to a connection between magnesium and gut microbiota [129]. Animal studies have demonstrated that magnesium supplementation can increase short chain fatty acid (SCFA) concentrations and enhance microbiota diversity [130]. On the other hand, variations in magnesium intake have been associated with changes in Bifidobacterium levels, tight junction protein expression, and cytokine activity [131]. Gender-specific factors can influence both magnesium metabolism and gut microbiota composition [132]. For example, estrogen has been shown to influence magnesium absorption and retention in women, potentially affecting the gut microbiota by modulating bacterial growth patterns [133]. During menopause, as estrogen levels decrease, the gut microbiota composition may shift, potentially influencing magnesium metabolism [133]. Furthermore, lifestyle differences between men and women, such as alcohol consumption, may also alter gut microbiota composition and magnesium excretion [134], leading to gender-specific differences in magnesium metabolism and absorption.

Both intrinsic and extrinsic factors significantly influence the bioavailability of nutrients from food sources. [135]. Furthermore, it is important to assess the actual nutritional contribution of specific foods within the framework of a healthy and balanced dietary pattern.

#### Dietary and Nutritional Factors Affecting Magnesium Bioavailability

Foods rich in non-fermentable dietary fiber typically contain high magnesium levels but exhibit low bioavailability, similar to iron. In contrast, fermentable or indigestible carbohydrates (e.g., inulin, resistant starch oligosaccharides, mannitol, and lactulose) enhance magnesium absorption [136].

Phytates and oxalates in high-fiber foods can reduce magnesium absorption through metal chelation. However, this effect is generally offset by the higher magnesium content in foods rich in phytates and cellulose [135,136].

Several minerals can influence magnesium absorption. High luminal phosphate concentrations hinder absorption via salt formation. Excessive calcium intake (over 10 mg/kg/day) reduces magnesium bioavailability [49], while dietary aluminum can decrease absorption fivefold, lower retention by 41%, and deplete bone magnesium levels [137]. Given the prevalence of aluminum in processed foods, it may be a significant factor in magnesium deficiency [49]. Similarly, high zinc intake disrupts magnesium balance; Nielsen et al. found that 53 mg of zinc/day (four times the recommended allowance) over 90 days reduces magnesium equilibrium [138].

Certain vitamins also play a pivotal role in magnesium bioavailability. Vitamin D promotes magnesium absorption and depends on magnesium for its activation and inactivation [139]. Similarly, vitamin B6 supports magnesium’s function in numerous enzymatic systems and enhances intracellular magnesium accumulation. A vitamin B6-deficient diet can lead to negative magnesium balance by increasing its excretion [140].

Casein or whey peptides may bind magnesium, potentially enhancing its absorption, similar to other cations [141]. Although low protein intake (<30 g/day) could negatively affect magnesium absorption, other studies suggest that magnesium utilization remains unaffected by protein intake levels [142].

Regarding beverages, magnesium levels are reduced by excessive ethanol consumption, non-alcoholic drinks, and coffee [143].

### 6.3. Dietary Patterns and Magnesium: A Gender Perspective

Magnesium intake is significantly influenced by dietary patterns, which vary based on cultural, lifestyle, and individual preferences [17]. A clear understanding of how these patterns influence magnesium status is key to formulating gender-specific dietary recommendations.

#### 6.3.1. Mediterranean Diet

The Mediterranean Diet (MedDiet) is a traditional dietary pattern originating from various cultures in the Mediterranean area. It includes a variety of foods, such as fruits, vegetables, fish, eggs, fermented dairy, grains, poultry, and limited amounts of red meat like lamb and beef. Extra virgin olive oil is emphasized as the primary fat source, and moderate consumption of red wine is encouraged [144]. Processed foods and sugary beverages are excluded from the diet. The MedDiet is characterized by a rich array of micronutrients and a favorable fatty acid composition, predominantly containing monounsaturated and polyunsaturated fats, with minimal saturated fats. It also includes magnesium-rich foods such as leafy greens, nuts, seeds, and certain fish, which contribute to its overall health benefits [144,145]. The MedDiet has been associated with overall metabolic and cardiovascular health, partly due to its nutrient-rich composition, including magnesium-rich foods [144,145]. Magnesium intake is thought to contribute to the beneficial effects of the MedDiet on overall physiological functions [146]. A recent study [147] explored gender differences in adherence to the MedDiet. The results showed that women had significantly higher adherence to the MedDiet. Additionally, women consumed more vegetables, fruits, legumes, and nuts—foods that are rich in magnesium—than men [147]. A recent meta-analysis [148] exploring gender differences in adherence to MediDiet interventions found that, although women showed higher adherence and lower dropout rates, these differences were not statistically significant. Further studies with gender-stratified data are needed to better understand these relationships. Nutritional recommendations based on the MedDiet should highlight the inclusion of magnesium-rich foods, with particular attention given to sex-specific guidelines.

#### 6.3.2. Plant-Based Diets

A plant-based diet consists of minimally processed fruits and vegetables, whole grains, legumes, nuts and seeds, herbs, and spices, while excluding all animal-derived products, including red meat, poultry, fish, eggs, and dairy [149]. These dietary patterns include variations such as vegan, vegetarian, and flexitarian diets, each differing in the degree of animal product exclusion. A systematic review demonstrates that vegans have the highest average magnesium intake (503 mg/day), followed by vegetarians (373 mg/day) and omnivores (302 mg/day). Both vegans and vegetarians tend to exceed the Estimated Average Requirement (EAR) for magnesium, while omnivores often fall short, particularly for men [150]. This suggests that plant-based diets, particularly vegan and vegetarian, may support adequate magnesium intake compared to omnivorous diets. A study comparing magnesium intake and status in pregnant women following plant-based versus Western diets found that those on plant-based diets had significantly higher magnesium intake (508 mg/day for vegetarians and 504 mg/day for low-meat eaters) compared to 412 mg/day in women on a Western diet [151]. Additionally, plant-based diets were linked to fewer calf cramps in the third trimester, supporting their role in improving magnesium status during pregnancy. However, phytates and oxalates in plant-based foods can bind magnesium, reducing its bioavailability [152]. Soaking, sprouting, or fermenting grains and legumes can help mitigate these effects.

#### 6.3.3. Western Diet

The Western diet is a modern eating pattern characterized by a high intake of processed foods, refined grains, red and processed meats, sugar-sweetened beverages, candies, sweets, fried foods, conventionally raised animal products, high-fat dairy, and fructose-rich products [153]. This dietary pattern provides low amounts of whole grains, fruits, vegetables, and nuts, which are key sources of magnesium. As a result, magnesium intake is typically insufficient, partly due to nutrient losses during food refinement. Epidemiological studies in Europe and North America have shown that individuals following a Western-style diet have a low magnesium intake, specifically <30–50% of the Recommended Dietary Allowance (RDA) for magnesium [154,155]. Research indicates that women are less likely than men to adhere to an unhealthy Western dietary pattern [156]. Moreover, the study’s findings suggest that men may be more susceptible to the effects of an unhealthy Western dietary pattern compared to women [157]. Gender differences in magnesium metabolism may also play a role, with some studies suggesting that women, despite consuming a similar or lower magnesium intake, may have a more efficient absorption or retention mechanism compared to men [45]. For instance, the high sodium content in the Western diet may exacerbate magnesium loss through increased urinary excretion in men [17]. However, the low magnesium content in the Western diet, combined with lifestyle factors, may influence magnesium status in both genders, particularly with aging.

**Figure 3:**
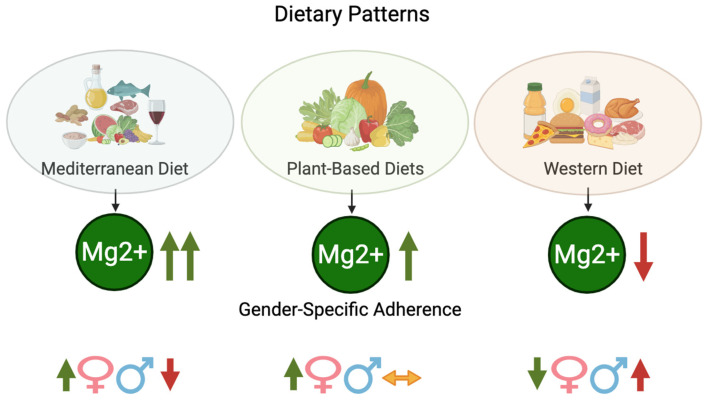
Dietary patterns and magnesium intake: Gender-specific adherence. Dietary patterns and their impact on magnesium (Mg^2+^) intake. The Mediterranean diet and plant-based diets are associated with higher Mg^2+^ intake, whereas the Western diet is linked to lower Mg^2+^ levels. Gender-specific adherence patterns indicate that women tend to follow Mediterranean and plant-based diets more than men, while men show higher adherence to the Western diet. The arrows indicate the relative amount of magnesium provided by each dietary pattern. Created in BioRender. https://BioRender.com/.

In conclusion, dietary patterns have distinct implications for magnesium intake and status, with gender-specific differences influencing absorption and retention mechanisms. As such, optimizing magnesium intake requires tailored dietary strategies that take these variations into account (Figure 3).

## 7. Dietary Strategies and Supplementation for Optimizing Magnesium Intake: A Gender-Perspective Magnesium Intake Through Diet and Supplementation

Magnesium intake often falls below recommended levels, particularly among individuals with suboptimal dietary habits. Gender differences significantly influence magnesium metabolism and requirements. Women, especially during the reproductive years, pregnancy, and menopause, may have higher magnesium needs due to hormonal changes that affect magnesium status. For instance, premenstrual magnesium depletion can lead to symptoms such as irritability and fatigue, while pregnancy increases magnesium requirements for fetal development. Postmenopausal women may experience changes in magnesium balance due to hormonal shifts that influence calcium and magnesium homeostasis.

In men, factors such as age, physical activity, and dietary habits also influence magnesium intake. As men age, their magnesium absorption efficiency may decrease, influencing overall magnesium balance. Older men may also experience changes in kidney function, which can impair magnesium regulation and lead to higher urinary magnesium loss. Furthermore, men with higher physical activity levels, particularly athletes, may have increased magnesium requirements due to greater losses through sweat and urine. Diets that are low in magnesium-rich foods, such as those typically high in processed foods, can further exacerbate magnesium insufficiency in this group. Additionally, variations in magnesium status have been observed in relation to metabolic and cardiovascular factors, particularly in men. To support adequate magnesium intake, a range of dietary strategies can be implemented, with a focus on incorporating magnesium-rich foods and enhancing absorption. These strategies should be tailored to meet the gender-specific needs of individuals.

1. Increasing Magnesium-Rich Food Consumption. Individuals following plant-based dietary patterns, such as the Mediterranean or vegetarian diets, typically exhibit higher magnesium intake compared to those adhering to conventional Western diets, which are often low in whole grains, fruits, vegetables, and nuts. These plant-based diets are naturally richer in magnesium, making them a beneficial strategy to improve magnesium status. The most straightforward method for enhancing magnesium intake is through the consumption of magnesium-rich foods. Leafy green vegetables (e.g., spinach, kale), legumes (e.g., beans, lentils), nuts and seeds (e.g., almonds, pumpkin seeds), whole grains (e.g., brown rice, quinoa), and certain fish (e.g., mackerel, salmon) are all excellent sources of magnesium. For example, 100 g of cooked spinach provide approximately 79 mg of magnesium, while 30 g (about one ounce) of almonds contain around 81 mg. Similarly, 30 g of pumpkin seeds can provide up to 150 mg, and 100 g of cooked quinoa provide approximately 64 mg of magnesium. Incorporating these foods into daily meals can significantly help meet the recommended dietary intake of magnesium.
2. Optimizing Magnesium Absorption. The bioavailability of magnesium in foods can be influenced by several factors. Phytates, found in whole grains and legumes, as well as oxalates, present in certain leafy vegetables, can bind magnesium and hinder its absorption. To counteract these effects, specific food preparation techniques can be utilized, such as soaking, fermenting, or sprouting grains and legumes. These methods help break down phytates and enhance the bioavailability of magnesium for absorption. Moreover, vitamin D plays a critical role in optimizing magnesium absorption in the gastrointestinal tract [139]. Ensuring sufficient vitamin D levels, either through sunlight exposure or dietary sources (e.g., fatty fish, fortified foods), can further improve magnesium intake and its overall bioavailability.
3. Addressing Magnesium Losses in the Diet. Magnesium intake can be compromised not only by inadequate consumption of magnesium-rich foods but also by factors that increase magnesium excretion. Diets high in processed foods, sugar-sweetened beverages, and excessive alcohol intake can lead to greater magnesium losses through urine. As a result, reducing the consumption of these foods and beverages is an important strategy in supporting magnesium retention and balance. Additionally, variations in digestive function may influence magnesium absorption, making dietary adjustments a useful strategy in optimizing intake.
4. Magnesium Supplementation. When dietary intake alone is inadequate, magnesium supplementation may be an option. Supplements are available in various forms, such as magnesium citrate, magnesium oxide, and magnesium glycinate, each differing in absorption rates and bioavailability. The choice of supplementation should be individualized, particularly for those with higher magnesium needs, such as older adults and individuals following restricted diets. Healthcare professionals can provide guidance on the most suitable forms and dosages to optimize magnesium intake.
5. Public Health Recommendations and Education. Public health initiatives and nutrition education programs play a key role in raising awareness about magnesium’s importance in nutrition. These efforts should aim to inform populations about the advantages of magnesium-rich foods, effective food preparation techniques to improve absorption, and the potential consequences of excessive magnesium loss. Furthermore, dietary guidelines should emphasize magnesium intake as an integral part of achieving overall nutritional adequacy.

## 8. Conclusions and Future Directions

Dietary strategies play a crucial role in optimizing magnesium intake, particularly for individuals with inadequate dietary habits. This review highlighted the essential role of magnesium in health, with a particular focus on gender differences in its metabolism and needs. Women may experience variations in magnesium status due to hormonal fluctuations, while in men, intake is influenced by factors such as age, physical activity, and overall dietary patterns. As men age, their magnesium needs may evolve due to physiological changes and lifestyle factors.

Individualized dietary approaches that consider gender-specific differences are essential. A one-size-fits-all strategy may not be sufficient to optimize magnesium intake, making it important to account for individual characteristics such as age, gender, and lifestyle. This personalized approach aligns with gender-based nutrition, which emphasizes the importance of integrating biological sex and gender in dietary recommendations.

Moreover, current dietary guidelines for magnesium intake may underestimate requirements when considering gender, age, and physiological states such as pregnancy, lactation, and aging. Emerging evidence suggests that magnesium needs may be higher in specific populations due to differences in absorption, retention, and metabolic demands. Therefore, refining dietary recommendations to better reflect these variables is crucial for ensuring optimal magnesium intake across diverse groups.

Future research should continue to investigate the complex relationship between diet, gender, and magnesium metabolism, with the aim of developing more precise and individualized dietary guidelines. Addressing these gender-specific needs in nutrition and public health policies will be essential for optimizing magnesium intake and supporting overall well-being.

## Figures and Tables

**Table 1:** Summary of gender-specific differences in magnesium metabolism, requirements, and health implications.

Mg^2+^ Actions	Women	Men	References
Hormonal Regulation	Estrogen enhances Mg^2+^ absorption and retention; levels decline after menopause, increasing deficiency risk.	Stable absorption; Mg^2+^ positively influences testosterone levels; deficiency may impair anabolic hormone production.	[21,93,95,109]
Life Stages Requirements	Increased requirements during pregnancy, lactation, and menopause; hormonal fluctuations affect Mg^2+^ homeostasis.	Higher needs among physically active men; aging reduces absorption efficiency and increases urinary loss.	[46,50,52,93]
Body Composition	More subcutaneous fat; menopause increases visceral fat; low Mg^2+^ linked to altered body composition and inflammation.	Higher visceral fat accumulation; inverse association between Mg^2+^ intake and waist circumference.	[15,16,84,86,87,107]
Metabolic Effects	Mg^2+^ modulates insulin sensitivity, especially during hormonal fluctuations; impacts premenstrual symptoms and metabolic stability.	Supports insulin sensitivity, glucose regulation, and testosterone balance; influences metabolic efficiency.	[31,58,70,89]
Bone and Musculoskeletal Health	Supports bone mineralization; deficiency post-menopause increases osteoporosis risk.	Essential for bone integrity and muscle maintenance; deficiency linked to sarcopenia in aging men.	[89,110,111]
Neurological and Emotional Effects	Regulates neurotransmitters, mood, and neuromuscular balance; involved in PMS, menstrual migraines, and dysmenorrhea management.	Contributes to neuromuscular function and muscle performance; Mg^2+^ status associated with muscle strength and functionality.	[56,69,80,88,99]

**Table 2:** Recommended dietary for magnesium according to different guidelines.

Gender	Group (Years)	LARN 2024 (Italy) [112] AIs	IOM (USA) [113] RDAs	EFSA (EU) [114] AIs
Men	7–12 months	80	75	80
	1–3 years	120	80	170
	4–6 years	150	130	230
	7–10 years	220	240	230
	11–14 years	290	240	300
	15–18 years	380	410	300
	19–69 years	350	400	350
	≥70 years	350	400	350
Women	7–12 months	80	75	80
	1–3 years	120	80	170
	4–6 years	150	130	230
	7–10 years	220	240	230
	11–14 years	290	240	250
	15–18 years	380	360	250
	19–69 years	350	310	300
	≥70 years	350	310	300
	Pregnancy	350	400–350 *	300
	Lactation	350	310–360 ^§^	300

Note: Magnesium requirements are expressed in milligrams per day (mg/day) for all values in this table. AIs, Adequate Intakes; RDAs, Recommended Dietary Allowances; * Pregnancy. 14–18 y: 400 mg/day; 19–30 y: 350 mg/day; 31–50 y: 360 mg/day. ^§^ Lactation. 14–18 y: 360 mg/day; 19–30 y: 310 mg/day; 31–50 y: 320 mg/day.

**Table 3:** Magnesium content in selected foods.

Category	Food Item	Magnesium (mg/100 g)	Magnesium (mg/Serving)	Standard Serving Size
Nuts and Seeds	Almonds	264	79	30 g
	Pine nuts	270	81	30 g
	Cashew nuts	260	78	30 g
	Peanuts, raw	210	63	30 g
Whole Grains	Quinoa, raw	189	189	100 g
	Oat, raw	177	177	100 g
	Wheat, durum	160	160	100 g
	Rice, brown, wholegrain, raw	116	116	100 g
	Millet, shelled	160	160	100 g
Leafy Greens	Spinach	60	120	200 g
	Swiss chard	81	162	200 g
Fish and Seafood	Shrimp or prawn	43	65	150 g
	Mackerel	21	42	200 g
Dairy Products	Low-fat yogurt	18	27	150 g
	Milk	11	33	300 mL
Chocolate and Derivatives	Dark chocolate (≥70%)	230	115	50 g

Note. According to the Food Composition Database for Epidemiological Studies in Italy, Version 1.2022 [119].

## Data Availability

Data sharing does not apply to this article as no datasets were generated or analyzed during the current study.
